# Evaluation of obstetricians’ surgical decision making in the management of uterine rupture

**DOI:** 10.1186/s12884-017-1367-8

**Published:** 2017-06-08

**Authors:** Justus Ndulue Eze, Okechukwu Bonaventure Anozie, Osaheni Lucky Lawani, Emmanuel Okechukwu Ndukwe, Uzoma Maryrose Agwu, Johnson Akuma Obuna

**Affiliations:** 1 Department of Obstetrics and Gynaecology, Federal Teaching Hospital, Abakaliki, Nigeria; 2 Department of Obstetrics and Gynaecology, Faculty of Medicine, Ebonyi State University, Abakaliki, Nigeria

**Keywords:** Uterine, Rupture, Maternal, Perinatal, Mortality, Surgical option, Obstetrician

## Abstract

**Background:**

Uterine rupture is an obstetric calamity with surgery as its management mainstay. Uterine repair without tubal ligation leaves a uterus that is more prone to repeat rupture while uterine repair with bilateral tubal ligation (BTL) or (sub)total hysterectomy predispose survivors to psychosocial problems like marital disharmony.

This study aims to evaluate obstetricians’ perspectives on surgical decision making in managing uterine rupture.

**Methods:**

A questionnaire-based cross-sectional study of obstetricians at the 46th annual scientific conference of Society of Gynaecology and Obstetrics of Nigeria in 2012. Data was analysed by descriptive and inferential statistics.

**Results:**

Seventy-nine out of 110 obstetricians (71.8%) responded to the survey, of which 42 (53.2%) were consultants, 60 (75.9%) practised in government hospitals and 67 (84.8%) in urban hospitals, and all respondents managed women with uterine rupture. Previous cesarean scars and injudicious use of oxytocic are the commonest predisposing causes, and uterine rupture carries very high incidences of maternal and perinatal mortality and morbidity. Uterine repair only was commonly performed by 38 (48.1%) and uterine repair with BTL or (sub) total hysterectomy by 41 (51.9%) respondents. Surgical management is guided mainly by patients’ conditions and obstetricians’ surgical skills.

**Conclusion:**

Obstetricians’ distribution in Nigeria leaves rural settings starved of specialist for obstetric emergencies. Caesarean scars are now a rising cause of ruptures. The surgical management of uterine rupture and obstetricians’ surgical preferences vary and are case scenario-dependent. Equitable redistribution of obstetricians and deployment of medical doctors to secondary hospitals in rural settings will make obstetric care more readily available and may reduce the prevalence and improve the outcome of uterine rupture. Obstetrician’s surgical decision-making should be guided by the prevailing case scenario and the ultimate aim should be to avert fatality and reduce morbidity.

**Electronic supplementary material:**

The online version of this article (doi:10.1186/s12884-017-1367-8) contains supplementary material, which is available to authorized users.

## Background

Uterine rupture stands as a single obstetric accident that exposes the flaws and inequities of health systems and the society at large due to the degree of neglect that it entails [1]. It is a common obstetric complication in low income countries [2, 3]. And affected mothers and their unborn babies suffer grievous outcomes, hence the various terms used to qualify uterine rupture in the literature [3–9]. The major predisposing factors are poverty, ignorance, illiteracy, traditional practices, high parity, poor infrastructure, cephalopelvic disproportion, previous uterine scars and poor obstetric care [1, 2, 4–7]. Poor obstetric care comprises lack of antenatal care, having unsupervised deliveries outside of health facilities, injudicious use of oxytocics to facilitate labour, and the resultant obstructed labour. All these factors abound in low income countries and make uterine rupture a commoner complication of pregnancy and labour compared to high income countries where uterine rupture very rarely complicates labour and the major risk factor is previous cesarean scar [2–4, 9, 10].

In Nigeria, uterine rupture is a frequent obstetric complication and reported incidence rates vary from 1 in 81 to 1 in 426 deliveries [2, 4, 5, 11–20]. These rates are largely similar to rates from sub-Saharan African countries like Ghana [1], Ethiopia [21, 22], Uganda [23] and Sudan [24], but generally higher than the rate of 1 in 445 deliveries from Tanzania [25]. Some studies show that most cases of uterine rupture occur outside the hospital. These consist of the 59 to 85% of women suffering uterine rupture who did not register for antenatal care (unbooked) [2, 4, 5, 12, 14–19] plus the proportion that registered for antenatal care (booked) but embarked on delivery outside the hospital and only return after the rupture had occurred. On the contrary, other studies show that greater than 55% of ruptures occur after the woman was admitted into a health facility [6, 26], highlighting the role of third delays in uterine rupture. Uterine rupture is often associated with high maternal and perinatal mortalities with reported maternal case fatality rates of 5.9 to 21.3% and perinatal mortality rates of 75.4 to 98.6% [2, 4, 5, 11–18, 20]. Maternal and perinatal morbidities are similarly high among survivors. Uterine dehiscence, uterine “windows” and occult or incomplete ruptures describe the partial separation of the uterine wall with intact overlying serosa [8, 16]. They are not often included as cases of uterine rupture because they seldom result in major maternal and fetal complications [16, 27].

Once a diagnosis of uterine rupture is made, surgery is the principal mode of management. The surgery often adopted is the quickest procedure that proves to be life-saving [13]. Available methods either conserve reproduction (uterine repair alone) or sterilise the patient (uterine repair with bilateral tubal ligation, subtotal hysterectomy or total abdominal hysterectomy) [1, 2, 4–7, 10–21, 28]. Preservation of the woman’s ability to reproduce by uterine repair alone leaves her with a uterine scar that has a higher risk of repeat rupture in future pregnancies [10, 17]. On the other hand, sterilisation by any of the other three surgical options makes the woman vulnerable to certain psychosocial complications linked to infertility, including marital disharmony [2, 5, 21]. Uterine rupture is the commonest indication for inevitable peripartum hysterectomy in Nigeria [29]. Despite these, there is wide variation in the frequency of use of the different surgical methods in managing uterine rupture in different centres and settings at different times both in Nigeria [2, 4, 5, 11, 12, 14–20] and other low income countries of sub-Saharan Africa [1, 6, 21–25] and in Asia [7, 13, 27, 30–32]. Thus, literature does not appear to favour any particular surgical method over the others. In fact, literature seems to suggest that the existing data are insufficient to advocate for any specific surgical method as the standard surgical management for uterine rupture [2]. But expectedly, obstetricians who are at the forefront in managing uterine rupture should have experiences to share. Hence, we deemed it pertinent to assess obstetricians’ overviews of surgical management of uterine rupture with a view to determine factors that guide their surgical decision making and evaluate their uses of, and experiences with as well as opinions about conserving and sterilising surgical methods.

The aims of this study are, therefore, to evaluate Nigerian obstetricians’ experiences with the surgical management of uterine rupture and their perspectives on the surgical management methods. A literature search did not find any similar study from Nigeria and/or elsewhere.

## Methods

This is a questionnaire-based cross-sectional study. The questionnaire consists of 17 items organised into two groups. Group A consisted of nine closed-ended questions on the participant’s age, religion, duration of practice, location of practice, and so forth, and group B comprised eight questions of which seven are closed-ended and one was open-ended (see Additional file 1). The questionnaire was pre-tested on 20 obstetricians (consultants and residents) of the obstetric department of the Federal Teaching Hospital, Abakaliki (FETHA), Nigeria, and then modified for clarity and correctness. The population studied were obstetricians who practice in Nigeria, and the study sample was randomly drawn from obstetricians in attendance at the 46th Annual General Meeting and Scientific Conference (AGM & SC) of Society of Gynaecology and Obstetrics of Nigeria (SOGON) held in 2012. Every obstetrician that attended the AGM & SC was eligible to participate if he/she were still in active practice and practised in Nigeria. Obstetricians who neither practised in Nigeria nor were in active practice, as well as those from the obstetric department FETHA, were excluded from the study.

Questionnaires were distributed to 110 respondents representing 37.9% of the 290 participants at the AGM&SC and 15.9% of the 692 obstetricians that were in active obstetric practice in Nigeria [33]. Data was collated and analysed using SPSS version 15, Chicago IL.

## Results

Seventy-nine questionnaires were analysed from 110 possible participants giving a response rate of 71.8%. The age range was from 28 to 69 years with a mean of 43.0 (± 8.3) years. The majority, 92.4%, were Christians, 53.2% were of consultant status (Table 1). For 10 years or less 57 (72.2%) of the doctors had been in obstetric practice, 60 (75.9%) only practised in government hospitals, 71 (89.9%) practised in tertiary hospitals and 66 (83.5%) practised in facilities located in urban communities (Table 1).

**Table 1:** Age groups, mean age, religion and professional status of obstetricians recruited for the study and participants’ duration, hospital, level and location of practice, *N* = 79

Item	N (%)
Age group	
≤ 30	4 (5.1)
31–40	23 (29.1)
41–50	36 (45.6)
51–60	11 (13.9)
≥ 61	5 (6.3)
Religion	
Christians	73 (92.4)
Moslems	5 (6.3)
Others	1 (1.3)
Professional status	
Consultant	42 (53.2)
Obstetric Residents	37 (46.8)
Practice duration	
≤ 10 years	57 (72.2)
11–20 years	13 (16.5)
21–30 years	6 (7.6)
31–40 years	1 (1.3)
≥ 41 years	2 (2.5)
Hospital owner	
Government	60 (75.9)
Private	7 (8.9)
Both government and private	12 (15.2)
Practice level	
Tertiary	71 (89.9)
Secondary	5 (6.4)
Secondary and tertiary	3 (3.7)
Practice location	
Urban	66 (83.5)
Rural	12 (15.2)
Urban and Rural	1 (1.3)

All the obstetricians managed uterine rupture of which 60 (75.9%) manage an average of 12 cases or less every year, while 12 (15.2%) and 7 (8.9%) respectively manage 13–24 and 25 or more cases annually.

The majority of the doctors opine that previous caesarean scar, injudicious use of oxytocic drugs and poor/no antenatal care in pregnancy are common risk factors for uterine rupture (Table 2). Seventy-five doctors (94.9%) opine that the association between uterine rupture and maternal mortality is high while the rest think it is low. Almost all doctors think that there is a high association between uterine rupture and maternal morbidity as well as with perinatal mortality and morbidity (Table 2).

**Table 2:** Obstetricians’ experience-based assessment of risk factors for, and associations between maternal and perinatal mortalities and morbidities and uterine ruptures, *N* = 79

Item		N (%)
Risk factors for uterine rupture		
Previous cesarean scar		51 (64.6)
Injudicious use of oxytocics		43 (54.4)
Poor/no antenatal care in pregnancy		43 (54.4)
Mismanagement of labour		30 (38.0)
Association between uterine rupture and		
Maternal mortality	High	75 (94.9)
	Low	4 (5.1)
Maternal morbidity	High	78 (98.7)
	Low	1 (1.3)
Perinatal mortality	High	79 (100.0)
	Low	0 (0.0)
Perinatal morbidity	High	79 (100.0)
	Low	0 (0.0)

Thirty-eight obstetricians (48.1%) commonly adopt uterine repair alone in managing uterine rupture compared 51.9% that rarely does, but the difference is not statistically significant. On the contrary, fewer obstetricians commonly use sterilising surgical methods compared to those that rarely do (Table 3).

**Table 3:** Obstetricians’ frequency of adoption of specific surgical options for managing uterine rupture, *N* = 79

Specific surgical option	Frequency	N (%)
Uterine repair		
(conservative surgical option)	commonly	38 (48.1)
	rarely	41 (51.9)
Sterilising surgical options		
Uterine repair with BTL^a^	commonly	28 (35.4)
	rarely	51 (64.6)
Subtotal hysterectomy	commonly	11 (13.9)
	rarely	68 (86.1)
Total abdominal hysterectomy	commonly	2 (2.6)
	Rarely	77 (97.4)

^a^ *BTL* Bilateral tubal ligation

The relationship between doctors’ age on one hand and their years of practice on the other and surgical options they adopt is not statistically significant (Table 4).

**Table 4:** Association between obstetricians’ age (years) and duration of practice (years) and use of a conservative surgery (A^*^) and sterilising surgeries (B^**^) in the management of uterine ruptures, N (%) = 79 (100.0)

Item	A^*^	B^**^	Fisher’s Exact Test	df	*P*-value
Age (years)					
≤ 30	1 (2.3)	1 (2.8)			
31–40	20 (46.5)	10 (27.8)	5.811	5	0.25
41–50	18 (41.9)	18 (50.0)			Not significant
51–60	3 (7.0)	6 (16.7)			
≥ 61	1 (2.3)	1 (2.8)			
Practice duration (years)					
≤ 10 years	30 (69.8)	27 (75,0)			
11–20 years	7 (16.3)	6 (16.7)	3.353	4	0.53
21–30 years	5 (11.6)	1 (2.8)			Not significant
31–40 years	0 (0.0)	1 (2.8)			
≥ 41 years	1 (2.3)	1 (2.8)			

A* - uterine repair alone

B** - uterine repair with BTL and (sub)total hysterectomy

Patient’s condition on presentation (87.3%), obstetricians’ surgical skills and ability to cope (84.8%), patient’s parity (78.5%) and the number of living children (77.2%), among others, are the main factors that influenced obstetricians’ decisions on surgical methods (Table 5).

**Table 5:** Factors that influence obstetricians’ decisions to adopt a specific surgical method in managing uterine rupture (doctors gave multiple answers), N (%) = 79 (100.00)

Item	N (%)
Patient’s clinical presentation	69 (87.3)
Physician’s surgical skill	67 (84.8)
Patient’s parity	62 (78.5)
Number of living children	61 (77.2)
Previous cesarean scar	42 (53.2)
Desire for more children	40 (50.6)
Maternal age	36 (45.6)
Marital status	35 (44.3)
Previous myomectomy scar	27 (34.2)
Socioeconomic status	19 (24.1)
Level of antenatal care	18 (22.8)
Biblical injunction	3 (3.8)

**Fig. 1:**
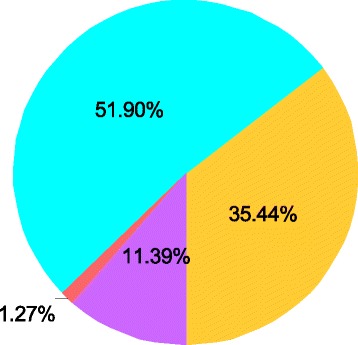
Obstetricians’ preferences of a standard surgery for managing uterine rupture; *N* = 79 (100.0%). (*Light blue*) Uterine repair alone (conservative surgery). (*Yellow*) Uterine repair with BTL* and hysterectomy** (Sterilising surgeries). (*Violet*) Both (conservative and sterilising surgeries). (*Orange*) None. *BTL = bilateral tubal ligation, **Hysterectomy = Total and subtotal hysterectomy

Assessment of obstetricians’ preferences for a standard surgical method for managing uterine rupture shows that 41 (51.9%) prefer sterilising surgeries, 35.4% uterine repair, and the rest either both or no preference (Fig. 1).

The commonest reasons for opting for conservative surgery by 28 obstetricians were high infant mortality rate (82.1%), the cultural significance of childbearing in stabilising marriage (75.0%) and use of modern contraceptive methods to forestall pregnancy (60.7%) (Table 6). On the other hand, poverty (90.2%), low patient educational status (90.2%) and poor health-seeking behaviour (85.4%) were the commonest reasons proffered by 41 obstetricians who opted for sterilising surgeries (Table 6).

**Table 6:** Reasons why obstetricians think that conservative surgery (uterine repair alone), *N* = 28, or sterilising surgeries (any of uterine repair with bilateral tubal ligation, subtotal hysterectomy and total abdominal hysterectomy), *N* = 41, should be adopted as standard surgery for managing uterine rupture

Reasons	N (%)
Uterine repair alone (conservative surgical option)	28 (100.00)
Infant mortality rate is high	23 (82.1)
Cultural significance of childbearing in marriage stabilisation	21 (75.0)
Use contraception to prevent further pregnancies if deemed risky and undesired	17 (60.7)
Counsel women with low parity properly	15 (53.6)
Literate mothers with low parity can be allowed to expand their families’ sizes	13 (46.4)
Circumstances of uterine rupture vary, so individualise treatment	13 (46.4)
Our people cherish their ability to reproduce	13 (46.4)
Infertility is a major cause of family strife	11 (39.3)
Counselling/informed consent should determine a woman’s assent to sterilisation	9 (32.1)
Preferred sex of babies may not have been achieved	8 (28.6)
Woman may be of low parity and desire more children	7 (25.0)
Ruptured uterus not likely to occur if subsequent pregnancies are well managed	7 (25.0)
Assisted reproduction is very costly	4 (14.3)
Because surrogacy and adoption are poorly established	3 (10.7)
Sterilising surgical options	41 (100.0)
Poverty	37 (90.2)
Low educational status of patients	37 (90.2)
Poor health-seeking behaviour and no antenatal care in pregnancy	35 (85.4)
High fertility rate, high mean parity and low contraceptive uptake	33 (80.5)
Very high recurrence rate	29 (70.7)
There may not be an appropriate hospital in her community	27 (65.9)
Most of the patients are in a bad state at presentation	27 (65.9)
Available hospitals may be in great disrepair	26 (63.4)
Poor access to tertiary hospitals and very high cost of care	26 (63.4)
Poor blood banking facilities	23 (56.1)
To remove the risk of recurrence and prevent death in future pregnancies	21 (51.2)
To prevent delay in presentation and management in subsequent pregnancy	19 (46.3)
Loss of faith in hospital services	17 (41.5)
To keep her alive	17 (41.5)
Other reproductive options such as surrogacy and adoption are available	9 (22.0)
Wounded womb is not very good for reproductive career	5 (12.2)

## Discussion

Uterine rupture remains an important cause of maternal and perinatal mortalities and morbidities in Nigeria and other low income countries, and thus a great source of public health concern [1, 2, 5, 16, 21, 23, 27, 28, 30, 32]. In resource-poor settings like Nigeria, uterine rupture is a reflection of ill-equipped, badly managed, and under-resourced health care systems that seem largely indifferent to the reproductive health needs of women [34].

Human resources for health planning, management and development have been strewn with crises in sub-Saharan Africa including Nigeria [35]. The finding in this study that the obstetric workforce is relatively young and disproportionately distributed in favour of government-owned tertiary health facilities in urban communities in the southern part of Nigeria is in line with Nigeria’s human resources for health country profile [35]. According to United Nations recommendations, the surgical resources of primary level facilities include the signal functions of Basic Emergency Obstetric Care (EmOC). Secondary level facilities also include Comprehensive EmOC [36]. A tertiary level facility should provide the highest level of surgery [35], with 24-h by 7 days safe anaesthesia and safe blood transfusion. In Nigeria, there are noticeable inadequacies at all three levels of health facilities such that most of the primary and secondary level and even some of the tertiary level facilities do not have the tools for required EmOC [36]. In addition, most obstetricians in this study work in tertiary hospitals located in urban communities, thus starving the primary and secondary facilities in rural communities of skilled staff. Moreover, most of the facilities do not have medical doctors [37, 38]. And contrary to what obtains in some other low income countries [6, 39, 40], associate clinicians are not employed to provide EmOC in Nigeria. Hence, poor illiterate women in rural communities and their unborn babies hardly get the needed attention [1] in pregnancy and childbirth, thereby increasing the prevalence of uterine rupture.

This study found that previous cesarean scar, injudicious use of oxytocics and unbooked pregnancies are the main predisposing factors for uterine rupture in Nigeria, thus corroborating other studies [2, 5, 12, 14–20]. So, doctors can actually reduce uterine rupture by not performing unnecessary caesarean sections, by the judicious use of oxytocic during pregnancy and childbirth, and by being extremely careful in the monitoring of women undergoing induction or augmentation of labour [6, 26]. This study also corroborates the findings by other studies that uterine rupture is associated with high incidences of maternal mortality and morbidity and even higher incidences of perinatal mortality and morbidity. Uterine rupture makes a huge contribution to maternal mortality in Nigeria [41].

Surgery is the mainstay of managing uterine rupture. Available surgeries are uterine repair on one hand, and uterine repair with bilateral tubal ligation (BTL) and hysterectomy (subtotal or total) on the other. This study found that whereas uterine repair alone was commonly adopted by about 48% of obstetricians, uterine repair with BTL, subtotal and total abdominal hysterectomies was adopted by 35, 14 and 3% respectively. This finding corroborates the results of some Nigerian studies [2, 4, 17] with a preponderance of uterine repair alone but varies with results of other studies [5, 11, 12, 14, 15, 17, 20, 22] in which sterilising surgical methods are preponderant.

Our study found that there is no statistically significant difference between obstetricians’ ages and their years of practice and the surgical methods they adopt in managing uterine rupture. There are factors, however, that assist obstetricians in surgical decision-making following uterine rupture. The commonest factors in this study are the patient’s condition on presentation, obstetricians’ surgical skills, patient’s parity and the number of living children, and the presence of previous cesarean scar. Local [2, 4, 5, 15, 16] and international [1, 13, 21] literature confirm some of these factors as very important in decision-making. Therefore, the surgery to perform following uterine rupture should depend on patient’s general condition, the extent of the uterine tear and surgeon’s ability to cope with the situation [13]. Thus, even if total hysterectomy is considered ideal for a case, a subtotal should be given preference because it will be quicker and life-saving [13].

More than 50% of obstetricians in this study prefer that sterilising surgeries be adopted as standard surgical management for uterine rupture, citing poverty, illiteracy, poor health-seeking behaviour and no antenatal care in pregnancy as the main reasons for their preference. Some authors share this opinion [13]. But 35% think otherwise citing high infant mortality, the cultural significance of childbearing in marriage stabilisation, use of contraceptives to prevent further risk and undesired pregnancies and proper counselling of women of low parity to make effective use of antenatal care as reasons to buttress their stand. Some 68% of these “repair only” respondents did not think that counselling or informed consent should determine a woman’s assent to sterilisation in the management of uterine rupture, a fact that requires further research. Still, another 1% think that the choice should be left open while 11% do not favour any surgical method at all, preferring to adopt the best method that appeals to the prevailing conditions on the instance of rupture. These findings corroborate the variations in surgical management of uterine rupture seen in the literature [1–5, 7, 11–25, 27, 28, 30–32]. They all appear to suggest that no method is ideal and that surgical method preference should be uterine rupture scenario-dependent.

## Conclusion

Obstetricians’ distribution in Nigeria is skewed in favour of the south as well as urban communities. And because most of the secondary health facilities lack medical doctors [37, 38] and associate clinicians [39, 40] are not employed to provide EmOC in Nigeria, rural-dwelling women and their unborn babies are starved of skilled attendance in pregnancy and childbirth. Common risk factors for uterine rupture abound in Nigeria and some like previous caesarean scars, are rising [42] and may ultimately cause a rise in the incidence of uterine ruptures from previous caesarean scars. Uterine rupture carries very high maternal and perinatal mortalities, and the morbidities among survivors can be lifelong and devastating. Its surgical management and obstetricians’ surgical preferences are scenario-dependent, influenced by patients’ condition on presentation, surgeons’ skills and ability to cope, and others. Obstetricians vary in their preferences for the standard surgical method, with reasons.

We recommend equitable redistribution of the obstetricians working in Nigeria to enhance the coverage of secondary hospitals, especially those secondary hospitals in or closer to rural communities. We also recommend the deployment of medical doctors trained in EmOC to provide services in the secondary hospitals, supervised by the obstetrician. And, if EmOC provision continues to remain quantitatively low, we recommend the employment of clinical assistants trained in EmOC to work under the supervision of the doctors [6, 39, 40] or the obstetricians. These measures will make EmOC more readily available and accessible to the poor and illiterate rural women who are at greatest risk of uterine rupture and ultimately lead to an improvement in the outcome of their pregnancies and childbirth with an overall reduction in the prevalence of uterine rupture. In the event of uterine rupture, we recommend that the obstetricians’ surgical decisions be guided by the prevailing case scenarios with the ultimate aims of averting fatalities and keeping morbidity extremely low.

## Additional files

Additional file 1: Questionnaire used for study. This questionnaire consists of 17 items organised into two groups. Group A consists of nine questions on respondent’s information and group B eight questions on the respondents experience with and opinion about surgeries used in managing uterine rupture. (DOCX 16 kb)

Additional file 2: Information for consent to respond. This document introduced the research topic and conveyed information to the responder on the researchers’ intention to conduct the survey, the way and manner the data will be handled and the possible beneficial use(s) to which the outcome after analyses will be put, and requested him/her to respond if he consented. (DOCX 12 kb)

## Abbreviations

AGM & SC: Annual General Meeting and Scientific Conference
BTL: Bilateral tubal ligation
CI: Confidence interval
FETHA: Federal Teaching Hospital, Abakaliki
IL: Illinois
SD: Standard deviation
SOGON: Society of Gynaecology and Obstetrics of Nigeria
SPSS: Statistical Package for the Social Sciences
