# Supplementary material for: Evaluation of obstetricians’ surgical decision making in the management of uterine rupture
Source: BMC Pregnancy Childbirth. 2017 Jun 8;17:179. doi: 10.1186/s12884-017-1367-8 (PMC5465586; doi:10.1186/s12884-017-1367-8)
Supplement: Supplementary file 1 — Questionnaire used for study. This questionnaire consists of 17 items organised into two groups. Group A consists of nine questions on respondent’s information and group B eight questions on the respondents experience with and opinion about surgeries used in managing uterine rupture. (DOCX 16 kb) [file 12884_2017_1367_MOESM1_ESM.docx]

Evaluation of obstetricians’ surgical decision making in the management of uterine rupture

Dear Respondent,

**Consent to Participate as a Respondent**

Uterine rupture is an obstetric condition.

The researchers wish to evaluate your surgical decision making in the management of uterine rupture using this questionnaire.

Data shall be anonymously collected and handled confidentially. Information derived from the data analysis and conclusions drawn therefrom may be published and help in advising clinicians and policymakers on strategies to further improve the surgical management and hopefully the outcome of uterine rupture.

If you give your consent to respond, please fill the attached questionnaire. It will take about 20 minutes.

Thank you,

**Research Team**

Questionnaire

Group A: Respondent’s information

1. Your age last birthday: _______ years
2. Your religion: Christian [ ], Moslem [ ], others (specify) _________________________
3. Your professional status: Consultant [ ], Obstetric Resident (Senior Registrar) [ ]
4. How many years have you been practicing obstetrics: < 10 [ ], 11-20 [ ], 21-30 [ ], 31-40 [ ], > 41 [ ]
5. In which country do you practice: Nigeria [ ], other (specify)________________

If you practice in Nigeria, continue.

1. In which region of Nigeria do you practice: North Central [ ], Northeast [ ], Northwest [ ], Southeast [ ], South South [ ], Southwest [ ]
2. Who owns the hospital that you practice in: Government [ ], private [ ], both [ ]
3. What level of service does your hospital offer: primary [ ], secondary [ ], tertiary [ ]
4. In which community is your hospital located: rural [ ], urban [ ]

Group B: Respondent’s experience with uterine rupture

1. Have you been involved in managing uterine rupture: yes [ ], no [ ]

If yes, continue.

1. What is the average number of uterine ruptures you manage each year: < 12 [ ], 13 – 24 [ ], 25 – 36 [ ], > 36 [ ]
2. In your experience, are the following risk factors for uterine rupture:
   1. Injudicious use of oxytocic: yes [ ], no [ ]
   2. Mismanagement of labour: yes [ ], no [ ]
   3. Poor/no antenatal care in pregnancy: yes [ ], no [ ]
   4. Previous caesarean scar: yes [ ], yes [ ]
3. In your experience, how is the association between uterine rupture and:
   1. Maternal mortality: high [ ], low [ ]
   2. Maternal morbidity: high [ ], low [ ]
   3. Perinatal mortality: high [ ], low [ ]
   4. Perinatal morbidity: high [ ], low [ ]
4. What is your frequency of use of the under-listed surgeries in managing uterine rupture:
   1. Uterine repair without bilateral tubal ligation (BTL): commonly [ ], rarely [ ]
   2. Uterine repair with BTL: commonly [ ], rarely [ ]
   3. Subtotal hysterectomy: commonly [ ], rarely [ ]
   4. Total abdominal hysterectomy: commonly [ ], rarely [ ]
5. Which of the under-listed facts, if any, help you decide the surgery for uterine rupture:
   1. Biblical injunction to multiply: yes [ ], no [ ]
   2. Maternal age: yes [ ], no [ ]
   3. Number of living children: yes [ ], no [ ]
   4. Obstetrician’s surgical skill: yes [ ], no [ ]
   5. Patient’s booking status: yes [ ], no [ ]
   6. Patient’s condition on presentation: yes [ ], no [ ]
   7. Patient’s desire for more children: yes [ ], no [ ]
   8. Patient’s marital status: yes [ ], no [ ]
   9. Patient’s parity: yes [ ], no [ ]
   10. Patient’s socioeconomic status: yes [ ], no [ ]
   11. Previous caesarean scar: yes [ ], no [ ]
   12. Previous myomectomy scar: yes [ ], no [ ]
6. Which of the following surgery(ies), if any, will you prefer to be the standard for managing uterine rupture: uterine repair without BTL [ ], uterine repair with BTL and/or (sub)total hysterectomy [ ], both [ ], none [ ]
7. What are the reason(s), if any, for the preference: (specify)________________________ ________________________________________________________________________ ________________________________________________________________________ ________________________________________________________________________________________________________________________________________________________________________________________________________________________________________________________________________________________________________________________________________________________________________________________________________________________________________________________________________________________________________________________

Research Team
