# Supplementary material for: Evaluation of obstetricians’ surgical decision making in the management of uterine rupture
Source: BMC Pregnancy Childbirth. 2017 Jun 8;17:179. doi: 10.1186/s12884-017-1367-8 (PMC5465586; doi:10.1186/s12884-017-1367-8)
Supplement: Supplementary file 2 — Information for consent to respond. This document introduced the research topic and conveyed information to the responder on the researchers’ intention to conduct the survey, the way and manner the data will be handled and the possible beneficial use(s) to which the outcome after analyses will be put, and requested him/her to respond if he consented. (DOCX 12 kb) [file 12884_2017_1367_MOESM2_ESM.docx]

Evaluation of obstetricians’ surgical decision making in the management of uterine rupture

Dear Respondent,

**Consent to Participate as a Respondent**

Uterine rupture is an obstetric condition.

The researchers wish to evaluate your surgical decision making in the management of uterine rupture using this questionnaire.

Data shall be anonymously collected and handled confidentially. Information derived from the data analysis and conclusions drawn therefrom may be published and help in advising clinicians and policymakers on strategies to further improve the surgical management and hopefully the outcome of uterine rupture.

If you give your consent to respond, please fill the attached questionnaire. It will take about 20 minutes.

Thank you,

**Research Team**
